# Bloody Marvels: In Situ Seed Saving and Intergenerational Malleability

**DOI:** 10.1111/maq.12684

**Published:** 2021-12-01

**Authors:** Katharine Dow

**Affiliations:** Department of Sociology, University of Cambridge

## Abstract

This article presents ethnographic research with in situ seed savers and seed activists in London. Unpicking the knotty relations between kinship, place, and generation among seed savers and their seeds, this article focuses on how the intrinsic and extrinsic get woven through generations and how the situ or environment of an entity is (and is not) recognized in its identity and multispecies kin relations. I argue that thinking about seeds and the worlds in which they grow suggests that they are not only embedded in their environments, but also embody their environments. If seeds bring with them their worlds, then they are inherently malleable, so seed savers are concerned about how commercial seed breeding and ex situ conservation denatures seeds’ embodied relationships with their environments and, with that, their inherent intergenerational malleability. [seed saving, multispecies kinship, generation, environment]

Seeds are a gift of nature, of past generations and diverse cultures. Thus, it is our inherent duty and responsibility to protect them and to pass them on to future generations. Seeds are the first link in the food chain, and the embodiment of biological and cultural diversity, and the repository of life’s future evolution.

——The International Commission on the Future of Food and Agriculture, *Manifesto on the Future of Seeds*

## Introduction

Londoners save plant seeds for many interrelated reasons, including to save money and share seed with others, conserve particular varieties, prevent biodiversity loss, ensure food quality and security, and resist the corporate enclosure of seeds by agribusiness (see also Kloppenburg 2000; Nazarea 2005). These motivations express a sense of intergenerational kinship with, and through, seeds, in which seed saving stands for the transmission of knowledge, connections with home, upbringing and tradition, a means of cultivating relationships with friends, kin, and neighbors, and of caring for plants and place. Cultivating such relationships through care may be particularly important for people living in a large, expensive, and sometimes onerous city like London.

Seed saving also reflects increased and widespread concern about the effects of climate change and biodiversity loss on future generations, which has led to inter-generational dynamics coming to the fore in contemporary environmental activism. A global activist movement around plant seeds has existed for many decades, mobilizing actors with different stakes in food production (see Peschard and Randeria 2020). In the United Kingdom, which has been at the heart of the development of industrialized agriculture, this movement links to other campaigns, including food sovereignty, anti-pesticide activism, agroecology, biodiversity conservation, and climate activism.^1^

I have been conducting ethnographic research on seed saving since 2015, initially looking at various sites in England, before deciding to focus on London. This has included participating in seed-saving events, carrying out auto-ethnographic seed-saving experiments, interviewing seed savers, and volunteering with relevant organizations. While London is a very modern, urban place, it has a large amount of green space, and people routinely seek out outdoor areas and grow plants, including for food. This is indicated by the years-long waiting lists for allotments in the city, which also reflects the fact that many people cannot afford garden space and social housing estates and apartment developments often do not provide growing space for residents. London is a city with a great diversity of residents, but also rampant inequality, which intersects with a hostile environment toward immigrants, systemic, and institutional racism (Erel 2018; Wardle and Obermuller 2019).

In this article, I focus on data gathered with people involved with London Freedom Seed Bank (LFSB), a small voluntary organization that keeps a library of seeds that have been grown by their growers’ network, as well as disseminating seeds and seed-saving knowledge for home and community gardeners interested in in situ^2^ seed saving in London. The leadership of LFSB^3^ is painfully aware of its whiteness and frequently discuss how they can improve representation of the city’s ethnic diversity in their constitution while avoiding tokenism. LFSB is, however, quite a loose organization that has room for many different levels of engagement and it is fair to say that their broader network is much more diverse, not only in terms of ethnicity, but also class, gender, and sexual identity and disability or health status. Several of those who currently lead and represent LFSB are also first- or second-generation migrants, including from former colonies of Britain like Ireland and New Zealand. These complex issues of positionality, representation, diversity, and inclusion will form the basis for a future article that explores the connections between seeds, people, and land in relation to “London seeds.” Here, my focus is on the ways seed savers think about and value intergenerational relationality and malleability between seeds and their environments.

As seed savers recognize, all the vegetal food we grow and eat is in some way the result of human intervention in the lives of plants, but activists express disquiet about the level of biological, economic, legal, and political control commercial seed breeders have over plant reproduction (see Barber 2019; ETC Group 2008; Peschard and Randeria 2020; Shiva 2016). They perceive these corporations to be invested in a very specific form of food production, primarily motivated by profit margins, rather than improving human health, conserving biodiversity, or responsible stewardship of the environment. In this article, I show that, as well as the loss and eventual extinction of non-commercial varieties and the biodiversity they represent and an antipathy to the values of the agribusiness world, London seed savers’ disquiet over human control of the constitution of biotic materials reflects a concern about the environments in which seeds are bred and grown, and how those environments go with them through their lives and into subsequent generations.

Donna Haraway has written that, “Seeds are brought into being by, and carry along with themselves wherever they go, specific ways of life as well as particular sorts of dispossession and death” (1997: 89); in other words, “nothing comes without its world” (1997: 37; Puig de la Bellacasa 2012). As other articles in this special issue discuss, the recent “environmental turn” in the biological sciences poses vital questions about the worlds in which things—whether they be human embryos, seeds, bacteria, stem cells, or lab mice—grow and what they bring with them as they move through subsequent generations (see also Jent 2019; Lamoreaux 2016; Lappé et al. 2019). As Margaret Lock (2017: 3) notes, this new attention to the environment in molecular science comes at the same time as geologists have reframed our current epoch as the Anthropocene, so, while biologists are seeing the effects of the environment on humans anew, geologists are recognizing the abysmal effects of human activities on the Earth.

The language of kinship, heredity, genetics, inheritance, and lineage in commercial seed breeding is notable in relation to the intergenerational theme of this special issue. Yet, the careful attention paid to seeds’ lineages in breeding programs detracts from the other kin networks in which seeds circulate (Aistara 2011; Bonneuil and Thomas 2010; Graddy 2013; Nazarea 2005: 9). This “gene fetishism” (Haraway 1997: 147) in commercial breeding has been criticized for backgrounding or erasing other aspects of seeds and their reproduction, including the embodied expertise and situated knowledge of growers, relationships, and exchanges between different growers, diverse, and localized farming practices and the cultural meanings of seeds (van Dooren 2008). Seed activists reject this gene fetishism. Their focus on seeds and the worlds in which they grow and how these are transmitted across both species and generations suggests that, for them, seeds are not only embedded in their environments, but also *embody* their environments.

In this article, I argue that when seeds are commercially bred, it is not only their relationships with people and place that are obscured (or, rather replaced with a different kind of relationship), but an aspect of what seed savers understand to be their nature—their inherent malleability, which comes through their capacity to embody their environments. This is important in how it speaks to current thinking in the biological sciences about the effects of environments across generations, but also because both campaigners and scholars including Haraway (2018) are using this ecological inflection point to call for alternative ways of making kin that resist the norms of the nuclear family as one response to the climate crisis (see also Dow and Lamoreaux 2020).

Andrea Ford (2019) has recently articulated embodied ecologies as “a conceptual framework for describing a fluidity between bodies and worlds that foregrounds relations instead of bounded entities,” in which “humans are inseparable from surrounding environments and also function as environments themselves.” In this article, I am describing how humans conceptualize plants, rather than humans (or parts thereof), though the fact that I am specifically discussing food plants suggests the folly of any kind of hard-and-fast distinctions between species. This focus on fluidity, porosity, and permeability in embodied ecologies is a fruitful means for thinking about how beings embody their worlds—and, as I will argue in relation to my own research, suggests how this makes beings, and their very natures, malleable.

As Elizabeth F. S. Roberts has discussed, the permeability of bodies becomes all too clear where people are exposed to toxic substances that, for example, make drinking Coca-Cola a survival strategy because tap water is unpotable. As Roberts puts it, “maintaining an inside and managing what enters it constitutes a crucial survival response within the continued violent capitalist interpenetration of all the earth’s biota” (2017: 594; see also Benezra this volume and Agard-Jones 2013). Parents are told that “breast is best” and that the first 1,000 days of a child’s existence are critical to their future survival (and the health of future generations) (Pentecost and Ross 2019), while many are exposed to residues of mercury and other toxins that bioaccumulate in breastmilk. These examples demand critical attention to the ways in which responsibility for children’s well-being tends to be leveled at mothers, even when parents have no good choices available to them because of the unequal ways in which the burdens of toxicity and environmental degradation are distributed— and thereby inherited across generations (Murphy 2011). As both Mollie K Murphy (2017) and Elizabeth Hoover (2018) point out, this makes such porosities and their effects matters of justice: reproductive justice, environmental justice and environmental reproductive justice. It also reminds us of the dangers of identifying causes and solutions to environmental issues in individuals (Lamoreaux 2019).

This article focuses on how the intrinsic and extrinsic get woven through generations in seed-saving practices and how the location, or *situ,* of an entity is (and is not) recognized in that organism’s identity and multispecies kin relations. It seeks to build on the recent conversations about body and environment outlined above, as well as Donna Haraway’s earlier characterization of gene fetishism and history of science literature on “pure line ontology,” by exploring the implications of a worldview in which seeds embody their environments. I will argue that, if seeds always bring with them their worlds, then there is an inherent malleability to them. This malleability is one of the qualities of seeds that seed savers are trying to preserve and why, for those interested in saving seeds for ethical and political reasons, in situ seed saving is vital for future generations.

## Kinship, Care, and Generations

At an event at Spitalfields City Farm in east London in autumn 2019, Helene, a White woman in her 20s who is a co-director of the London Freedom Seed Bank, gave a short talk. She spoke about the contrasting philosophies and practices of commercial seed breeding and in situ seed saving and the dependency that agribusiness promotes through forcing farmers to buy seed each year, as well as how growing a small number of commercially produced varieties leads to a loss of genetic diversity and, with it, flavor. Helene said that seeds ought to be “reframed” from the industrial model of ownership toward being a “public good,” while noting that it is “sad that this reframing is required.” After all, she said, “it comes down to care. We’re not consumers, but stewards, keepers and guardians, looking after seed for the future and each other” (see also Schulze 2020).

Like this special issue’s editors, I take a capacious approach to kinship, seeing it as encompassing the kinds of experiences of relationality and practices of caregiving that are illustrated by Helene’s comments above. Commercially produced seeds are an example of what Haraway (1997) describes as “proprietary kin”: they are valued for their genes while the natural-social articulations through which they are produced are disavowed. By contrast, saving, sharing, and swapping seeds can express a sense of kinship with and through plants, environments, and other humans. A key difference in these two ways of approaching seeds is the extent to which beings are conceptualized and valued (only) as individuals.

Charlotte, a White woman in her 30s, is another co-director of LFSB who also works as a professional gardener. She described how she felt when sorting seeds to go into LFSB’s library:

[Seeds are] just such beautiful objects and full of so much potential. I love the, one of my favourite things about doing the seed bank is sorting the seeds, because it’s one of the most mundane things, and boring things, but also where you really feel the relationship between me and the seeds, or us as humans, and the seeds, and it feels like you’re really doing something so ancient, something so important, but so simple and so neglected. Just deciding which seeds to save, deciding which seeds you want to bestow on the next generation, or keep alive for the future. Such a simple but important act. And that’s just massively inspiring.

—(Charlotte Dove, interview, February 12, 2019)

Charlotte was six months pregnant with her second child at the time of our interview and, while she does not draw explicit parallels between children and seeds in this quote, many other interviewees (whether parents or not) have likened seeds to babies and children. In particular, seeds seem to remind people of children because of their need for care. Many have described how they care for their seedlings by preparing soil and compost mixes, ensuring they have enough light, water, and space. Charlotte’s description here also captures the caring labor of seed saving—it is mundane and boring, but is also about feelings, relationships, and doing something “ancient.”

Seed saving reminds people of parenting or caregiving in more or less literal ways, reflecting the broad cultural resonance of kinship and reproduction. For research participants, seeds represent life, growth, fertility, and even magic. Simon, a young Black man, for example, said:

The seeds of today are the flowers of tomorrow. The flowers of tomorrow are in the seeds of today. That’s what seeds mean to me. There’s so much power within a seed. Us as humans, I know it might not relate, but our mothers and fathers, there was a seed into an egg, similar principle, and out came life abundance. We have life, we have so much to be appreciative for. It’s just the same with a flower. Within that one seed, if that seed is put in the right conditions, soil, light, water, that seed is going to germinate. And depending on what species or genus that seed is, it’s going to either create flowers for bees or fruits for insects, animals and humans to eat. The power within a seed is exponential. The ramifications are too much. Seriously, it’s like, when I take seeds and I’m planting, it’s just, I can’t explain.

—(Simon Parks, online interview, June 11, 2020)

Many participants also involve children in their growing and seed-saving practice, whether children in their own families, local children, or children they work with at schools or community gardens. LFSB steering group member Richard, a White man in his 40s, has taken on the task of sorting and packaging seeds to send to growers in 2021, assisted by his daughter and some measuring scoops they have crafted from her Playmobil set.

Simon works with his neighbor Babu to grow food and save seeds in some communal beds on their council estate. Babu, a man in his 40s of Indian descent, described how this activity had helped cement a strong friendship with Simon and how he sees growing food together and sharing knowledge about how to do so as a vital way to grow his local community. During our online interview, his son wandered into the room, prompting a conversation about involving children in gardening. Babu said:

[W]e all loved it as kids, scrambling around overgrown gardens and whatever, parks, picking up blackberries. So that’s the basic strategy I employ. My thinking is, when a child picks off a fruit or off a plant, and eats it, I mean, we all know the deep connection that we have from that, from our childhood. So, getting them doing that, and then just introduce them to the rest of the growing. So, Simon and I are constantly inviting any partially interested passer-by to come and talk to us, “look at the plants, look at what we’re doing.” Always friendly to everyone, especially if they’ve got kids. That’s great. Bring in the kids, get them to eat it. Give them some cuttings to take away or give them some little sprouting plants, strawberries, or tomatoes or oregano, thyme, etcetera, to take away.

—(Babu Roy, online interview, June 17, 2020)

Here, Babu points to the importance of community, which, like kinship, is guided by principles of care, sharing and togetherness. Growing food from saved seeds, and sharing the knowledge of how to do so, can create intergenerational bonds between people.

A majority of my interviewees have mentioned first becoming interested in growing food as a result of observing and helping their parents or grandparents grow food and save seeds. Sam, a White woman in her 40s, told me that she had learned to save seeds from her grandfather, “a very down-to-earth Yorkshire man” and told me that she thought saving seeds was “just what everybody did.” For participants like her, seeds therefore encapsulate intergenerational knowledge, practice, and relationships. This is all the more pertinent when we consider the life cycle of seeds: most of the plant seeds that circulate through LFSB are annuals, so produce new generations each year they are grown. These new generations might then be saved and grown by the same person, or shared or swapped with others, in the new conditions that each year, space or grower brings. Saving and sharing seeds thereby embeds people in intergenerational relationships of care with plants, environments, and other humans.

## Valuing Flavor, Savoring Values

Saving seeds is a way to resist and circumvent mainstream food systems for political and gustatory reasons. Many people save seeds as a way to produce crops that are difficult to find or expensive to buy, but which they like to eat. There is an established movement to preserve heritage or heirloom varieties in the United Kingdom (Curry 2019; see also Hoover 2017; Nazarea 2005), but this is not a particular interest of the people I have met in London. Instead, saving seeds allows these growers to select those varieties that they know grow well in their local conditions and that they enjoy eating, as well as to disengage from industrialized agriculture and supermarket consumption.

Tomatoes are a popular crop among London seed savers because they are relatively easy to grow and save seeds from and they are a staple ingredient in innumerable cuisines; further, tomatoes sold in supermarkets typically lack flavor. The Galina cherry tomato is a favorite variety in the LFSB’s library, valued for its flavor and its ability to produce many fruits even when grown in pots. This is an important consideration for growers in London, where growing space is at a premium. The first Galina seeds in the LFSB’s library seem to have originated with a donation by Patrick McCabe, who had saved them from a batch he acquired from Real Seeds, the pioneering Welsh seed-saving organization in the United Kingdom, from which several participants source seeds.

Patrick is a White, middle-aged, neurodiverse gay man who grew up in New Zealand. He said that he noticed, after moving to the United Kingdom, that supermarket vegetables tended to lack flavor, which led him to find out more about industrialized food systems. Patrick values flavor as an enthusiastic home cook and as a way to take care of himself, as someone who is recovering from mental health and addiction issues. He explained:

[I]t’s widely accepted that the nutritional quality in a lot of the commercially available fruit and vegetables is much less than what it used to be. So, here we are in a society where people are buying all these vitamins and mineral supplements that normally should’ve been available from a really diverse fruit and vegetable, grains, nuts and pulses that is all obtainable from that, largely. So, why are we all buying that? And people are buying it because a lot of the available stuff isn’t producing that and isn’t giving those nutrients. Now, that can be issues to do with soil, but also it’s an issue to do with breeding and the plants that are being selected and the way they’ve been bred isn’t providing the same kinds of benefits.

—(Patrick McCabe, online interview, October 22, 2020)

Growing food from saved seeds allows people to access a broader range of foods, which can help them make meals that are tastier and cheaper, but which also represent a connection to home, family, or one’s childhood (see also Heitlinger 2017). For Patrick, this was about comparing produce in the United Kingdom with the quality of food from his parents’ smallholding and in the markets of his native New Zealand. For Noriko, a chef who grew up in Japan observing her grandmother growing food in the countryside, saving seeds allowed her to grow ingredients like shiso leaf, which is expensive to source in the United Kingdom. For Sam, whose family come from Yorkshire, it was a way of getting her autistic son, who refuses to eat supermarket vegetables, to eat home-grown leafy greens. These connections to home and family are important, whether or not seed savers are first- or second-generation migrants to the United Kingdom, adding another layer to the ways in which growing food from saved seeds is also a form of intergenerational kinship practice.

Valuing flavor relates not only to connections between people and plants, or people and place, but also between plants and place. The close relationship between flavor and location in in situ seed-saving brings to mind the concept of *terroir*. Amy Trubek writes that terroir reflects a particular attention that French people pay to ‘the role of the natural world in the taste of food and drink’ (2008: 19). She explains, “When the French take a bite of cheese or a sip of wine, they taste the earth: rock, grass, hillside, valley, plateau. They ingest nature, and this taste signifies pleasure, a desirable good” (2008: 19–20). As Heather Paxson discusses (2013), concepts like terroir may be vital marketing tools for small-scale artisanal producers with tight profit margins. Such products may seem more authentic, rustic, and personal, but are also located in place; for artisanal cheese makers, this contrasts with the quintessential “American cheese,” produced with milk from mega-dairies. This resonates with the distinction my participants make between shop-bought and home-grown produce—and with the distinction between saved and commercially bred seed. Most importantly, for the purposes of my argument here, is the possibility that terroir raises for a food, through its flavor, to bring with it the world in which it has been made. It seems that something similar is happening here in the idea that food grown from saved seeds tastes better, that the world in which it has grown can translate on the tongue of its consumer, and thereby become part of them, too (Butler 2019; DuPuis 2015: 139).

## Purity, Diversity, and Malleable Boundaries

Selecting plants for seed saving is a balance between keeping the genetic diversity, and eliminating undesirable characteristics (“roguing” the crop) to keep the variety true-to-type, and this is something that becomes easier with experience.

—Sue Stickland, *Back Garden Seed Saving*

**Figure 1:**
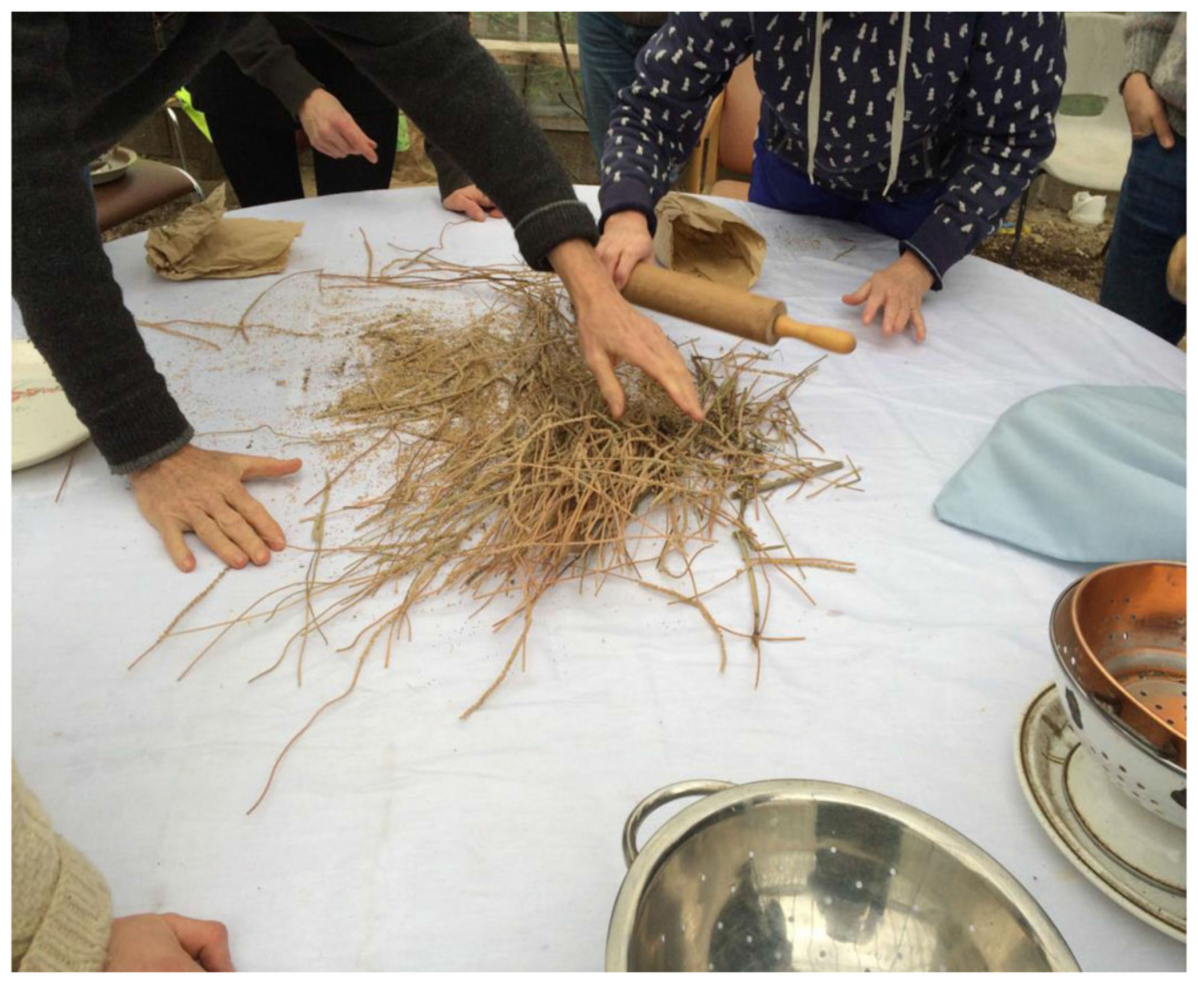
Seed-saving workshop, February 2016, OrganicLea, east London. (Photo by author) [This figure appears in color in the online issue]

At many of the talks I have attended at seed saving events, F1 seed, as the epitome of commercial seed breeding, has been presented as if it were a wraith haunting people’s gardens and allotments. F1 (Filial 1) crop varieties are produced through crossing two parent varieties that have been bred and selected for particular characteristics to make the resultant crop uniform. They usually have hybrid vigor, which means they are more vigorous in the first generation, but if saved, they do not produce “true-to-type,” so they lose their uniformity in the next generation. Contrasting the commercial seed and the saved and/or open-pollinated seed can be a potent rhetorical strategy for seed activists, (see also Franklin et al. 2000; Shiva 2016). However, as the epigraph above suggests, it does not necessarily reflect the malleability of the boundaries between culture and nature, purity and diversity, reliability and unreliability that characterize in situ seed-saving practice. Traits can be bred in and out through different generations and when I attended a seed-saving workshop at OrganicLea in east London early in my research (see Figure 1), I learned that if a seed produced under non-organic conditions is grown out for a generation using organic methods, in the next generation, it is considered to be organic.

During a meeting of the LFSB growers’ network in February 2020, someone asked whether, if he and his friend (who was also present), who both live in the Streatham area of south London, took some of the same variety of tomato seeds to grow out and donate back to the seed bank, then, would those tomatoes become “too Streatham”? Charlotte replied that they should not worry because they are aiming to build up London generations, thus smoothing Streatham out into the larger city of which it is part. Richard added that, because plants grown in London will always be proximate to other plants, they can never be guaranteed to be isolated, implying that these varieties grown separately but close together would be unlikely to achieve any sort of genetic purity. He suggested that one way to deal with this uncertainty would be to rename varieties that have unclear parentage, for example naming them after the allotment in which they were grown—thus locating their identity in their situ. When I interviewed Richard the following year about how he has used the software Airtable to curate the seed bank, he reflected on the level of “granularity,” as he put it, that might be worth recording. He wondered out loud where to draw the line of locality: at London or at individual gardens? He asked, “Are we going to get to the stage where we say, well this is a South London True Brandywine [tomato], or a garden True Brandywine and therefore it’s intrinsically different to the one that is grown in a different garden?”

The co-directors of the LFSB are concerned about seed-saving knowledge being lost to younger generations. Without knowledge of how to save seeds, the seeds themselves will also be lost. This knowledge has several layers, from the names and properties of particular varieties, to understanding how different plants are pollinated, to how to harvest, store, and germinate particular seeds and, perhaps most importantly, how specific varieties respond to particular conditions. Naming has an important place in the history of biology, in relation to taxonomy, the science of classifying and naming biological organisms based on shared characteristics (see Subramaniam 2014: 77). Latin names relate to taxonomic ontologies and the formalities of botanical science, while “common names” reflect the vagaries of human memory and a valuing of plants’ situated relationships with people and land (Sonjasdotter 2018: 314). London seed savers value knowledge about seeds, but less to order the world and more to facilitate productive growing and a sense of community. This could be partly attributed to the fact that most are amateur gardeners and are not reliant on their crops to survive, but this is also about their commitment to an alternative politics, in which plants are (re)situated in relation to their environments and multispecies relationships.

Historians of science have characterized the “pure line” model of heredity that became influential in Europe during the 19th century and into the early 20th century, which is essential to industrialized models of seed breeding and production. Bonneuil and Thomas write, “The ontology of ‘genetic modernism’ considered living beings as having an intrinsic genetic identity, sealed off from the vagaries of the environment, and favored serial and stable forms of life, which were achieved through the production of plant populations composed of isogenotypic individuals (clones, pure lines, F1 hybrids)” (Bonneuil and Thomas 2010: 532). This pure line ontology responded to a new demand for economies of scale and industrial rationalization that valued purity, stability, and uniformity. By the turn of the 20th century, “Purity was no longer the product of history, genealogical stories, personal knowledge, and controlled interpersonal relations, but rather a structural property, engraved in the genotype (i.e., homozygosis). Purity thus lost its domestic dimension” (2010: 539). Ironically, once plants came to be bred in isolation from their environments, they became conceptualized as stable individuals that could be used to measure changing environments and processes of adaptation (Bonneuil and Thomas 2010: 541). Bonneuil and Thomas describe this kind of thinking as “phyto-eugenic” and Banu Subramaniam (2014: 67) has written about how the pure line ontology of plant breeding fed into eugenics (in humans) via genetic and evolutionary science. As Subramaniam says, eugenics is only thinkable if we believe that individuals are largely or solely the product of their genes; this shows what is at stake when genetic purity is fetishized and when individuals are isolated from multispecies connections with their environments.

The seed savers I know in London are less concerned with conserving the purity of particular varieties and more interested in preserving biodiversity more generally, through fostering a human community that grows, saves, and shares a diverse range of plant varieties. This in situ approach is about allowing for different varieties of seeds, as well as different environments for those seeds to grow in, for the benefit of the plants, the people who grow them, the environment, and other species such as pollinators too. This is in contrast to the commercial seed, which is bred for uniformity and stability so that it can be mono-cropped in different locations and gradually edge out other varieties, which thereby reduces global biodiversity.

T. Garrett Graddy, writing about potato conservation in the Peruvian Andes, argues that plants, people, and place are closely entangled: “In situ describes collective adaptation to, with, and of the ecological site at hand—a situation that is continually changing and regenerating itself anew. From the perspective of in situ, place serves as the site of interdependence, interaction, and reciprocity—among humans and between humans and non-human entities” (2013: 441). Importantly, as she points out, situating conservation or seed saving in specific locations or traditional practices does not mean that these relationships or beings are static. Unlike ex situ seeds that have in a sense been preserved in time and space, in situ seeds embody the capacity for change.

**Figure 2:**
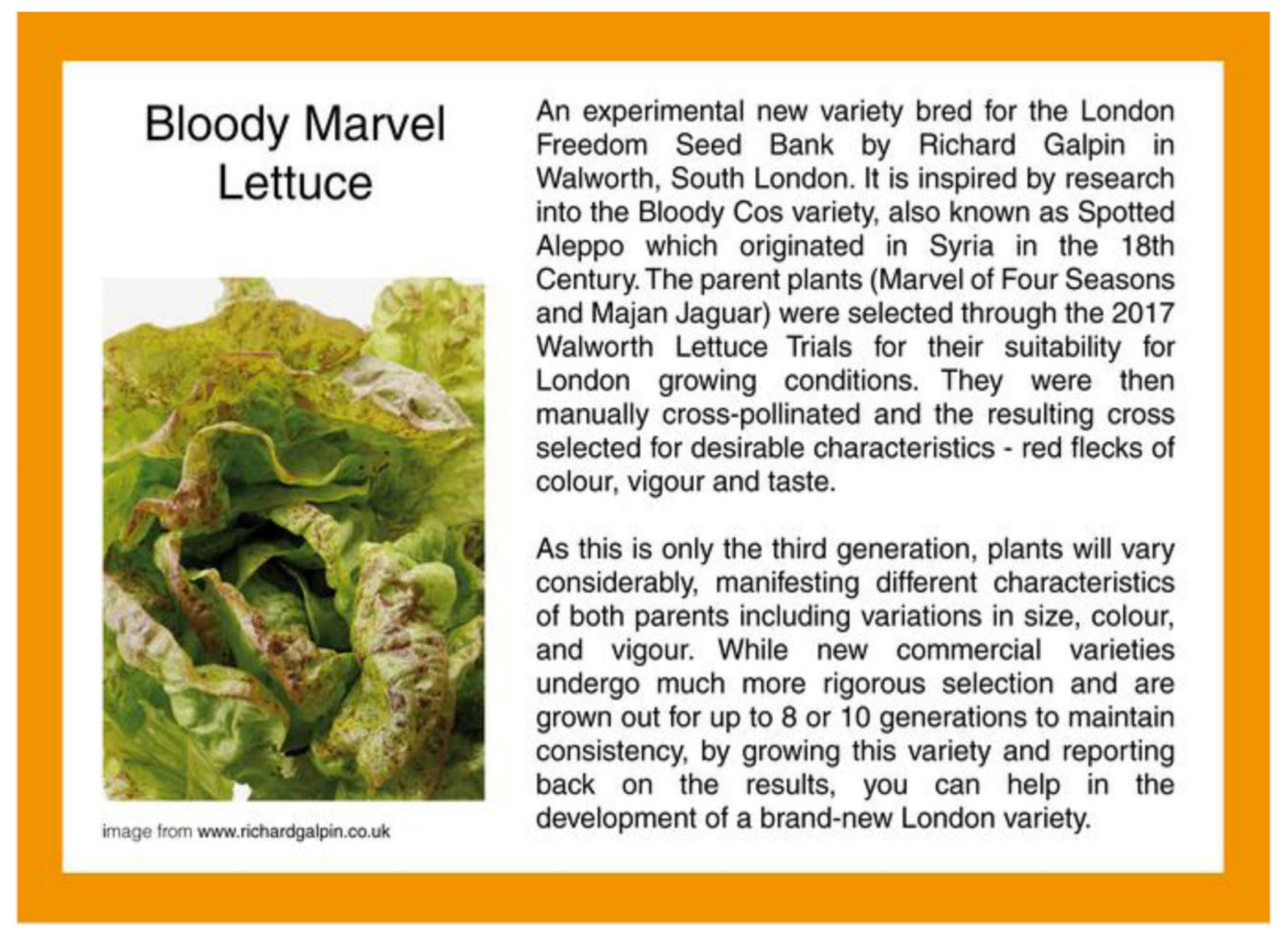
Bloody marvel lettuce postcard designed for LFSB by Sara Heitlinger and Franc Purg. [This figure appears in color in the online issue]

Initially, I had assumed that London seed savers might be hesitant about actively breeding seeds themselves, as this might seem too like what commercial seed breeders do, but, in fact, for some, breeding new locally adapted varieties can even add to biodiversity. Richard is an enthusiastic lettuce breeder, who has developed the bloody marvel lettuce (see Figure 2). When I visited him at home in south London to record an interview in 2019, he first took me on a tour of his small garden, where I saw his lettuces growing for myself. As Figure 2 shows, this is an experiment in generation, in more than one sense of the word. As the postcard explains, in the third generation, “plants will vary considerably, manifesting different characteristics of both parents.” This is contrasted with new commercial varieties, which “are grown out for up to 8 or 10 generations to maintain consistency.” Such consistency is assumed to be a desirable characteristic by commercial breeders. On the postcard, however, it is implicitly contrasted with the more experimental and collective endeavor of different Londoners helping develop bloody marvel together.

During the United Kingdom’s first Covid-19 lockdown, bloody marvel was the topic of a months-long conversation in the LFSB growers’ network WhatsApp group. In early April 2020, one member, who also works as a professional gardener and educator, posted in the group asking for advice on “roguing out” his bloody marvel. Roguing is the process of removing crops with undesirable characteristics from a crop; with plants being grown for seed this is particularly important for preventing those traits being passed on to future generations (Stickland 2014: 191). Richard responded with detailed advice, referring back to the breeding process, remarking that he thought another variety had emerged and suggesting it would need a new name. The conversation continued, illustrated by photos of the minute variations between lettuces that were, in Richard’s view, true to bloody marvel and those that, despite having the same parents, were not. In this way, the name bloody marvel, which was chosen to reflect its parentage and its red-speckled appearance, has become ever more apt, as its (genetic) identity is marvelously labile, with traits bleeding in and out of the generations.

These considerations of culture and nature, purity and diversity, and reliability and experimentation bring me to Marilyn Strathern’s theorization of “English” kinship at the turn of this millennium. Strathern (1992b) points out that in the modern(ist) model of English kinship, individual parents come together to produce children who, although constituted by them, are uniquely different from their parents. Each new generation produces unique individuals, and thereby more diversity, in the world. In this way, time, biogenetic substance, and identity seem to flow irreversibly downward and diversity accretes over time. This does not entirely translate across species boundaries, not least because Euro-Americans hesitate to think of plants as individuals in the same way as people. However, seed savers do value something like the accretion of diversity that comes with new generations of humans described by Strathern when it comes to plants. The example of the bloody marvel shows that what London seed savers are aiming for is not the uniformity of the strictly defined and controlled commercial seed, but abundant diversity, continuing or extending across generations. Breeding new varieties oneself offers the opportunity to go beyond conservation, by *adding to* the world’s biodiversity.

## Seeds and Their Worlds

Saving seeds is a low-tech process of harvesting seed from plants by hand—or sometimes by mouth, when seeds are separated from plants by blowing—and with the help of noticeably domestic tools like sieves and rolling pins, storing them in paper bags or glass jars, in a shed, cupboard, or the fridge, and then planting them out, giving them away or swapping them for other seeds with neighbors, friends, family, or participants in a seed swap. This seed saving is both in situ and situated. Research participants talk about how seeds grow well in particular places, because of (micro)climates, soil, local ecologies, pollution, insect populations, and so on, but also because of their relationships with, and dependency on, people. By contrast, in the laboratories of commercial breeders, plants are ideally kept in conditions of utmost biological, and commercial, control, in very different contexts from how they might grow on a garden or farm, let alone in the wild. My participants’ practice also contrasts with the ex situ approach of institutions like the Millennium Seed Bank Partnership, the largest ex situ seed collection in the world. Such seed banks use cryopreservation and specially engineered equipment and buildings that prioritise the preservation of genetic traceability and biological audit trails (see Chacko 2019b). As Kay Lewis-Jones (2019) writes, ex situ conservation cannot conserve the contexts in which plants grow, so is better suited to a “post-natural” future.

Field philosopher Thom van Dooren (2009) thinks ex situ gene banks have a place in conservation but argues that they need to be complemented by more support for in situ initiatives and greater work to conserve biological and social diversity in agricultural environments (see also Graddy 2013). Van Dooren criticizes the precept within commercial seed breeding that such seeds are “invented.” By foregrounding invention, he argues, “a rupture is effected which denies the seed’s embeddedness in broader webs of meaning and life” (2008: 683). Drawing on Haraway’s work, he suggests shifting the focus away from invention towards emergence: “As emergents, seeds arise out of, but remain within, situated contexts and environments. There is no rupturing in emergence. Rather, seeds (and plant varieties) germinate, grow, die, and evolve within ongoing networks of coconstitution in which diverse agencies play diverse roles” (2008: 689).

Marilyn Strathern’s work has provided intergenerational inspiration to both Donna Haraway and Thom van Dooren when thinking about (multispecies) relationality—as well as my own. Strathern pointed out nearly 30 years ago that the assumption that people are influenced by their environment as well as their genes betrays a sense that an individual is a whole, substantial and visible entity, while their environment, which seems to exist in the abstract, exists beyond them (1992a: 123). She wrote that, “It is an anthropological axiom that however discrete they appear to be, entities are the products of relations; nothing is not *embedded in* some context or worldview that gives it its special shape” (1992b: 12, emphasis added). Haraway, meanwhile, has written,

A seed contains inside its coat the history of practices such as collecting, breeding, marketing, taxonomizing, patenting, biochemically analyzing, advertising, eating, cultivating, harvesting, celebrating, and starving. A seed produced in the biotechnological institutions now spread around the world contains the specifications for labor systems, planting calendars, pest-control procedures, marketing, land holding, and beliefs about hunger and well-being. [1997: 129]

While Strathern and van Dooren’s observations describe cultural formulations in which an individual and its environment are “coconstituted,” these formulations nonetheless describe individuals as embedded within their environments, and so still in some way discrete from them. What I have found among London seed savers in the 21 century, however, is a rather more porous understanding of the boundaries between entity and environment. If seeds carry their worlds along with them in their coats, as Haraway suggests, their worlds are intrinsic to them. This not only dissolves the boundary between the individual and their environment, but also inverts the usual relationship between individual and environment, as the environment is taken into the individual rather than the individual being embedded within its environment.

Running through these questions about purity and diversity, individual and environment, is a set of binary contrasts, which may be mobilized for rhetorical and political purposes. The example of the bloody marvel lettuce shows that, in practice, London seed saving is more about diversity and experimentalism than purity. Andrea Ford argues that the embodied ecologies framework prioritizes relations over entities and rejects Cartesian dualisms. I agree that this is important, perhaps especially in our current political climate and ecological predicament, but dualisms can still be valuable in communicating instances of injustice and inequality. This tricky tension is illustrated by a debate between Donna Haraway and Vandana Shiva, the figurehead of the seed-saving movement and an inspirational figure for LFSB, over transgenic organisms. Writing in the 1990s, Haraway sensed a primordial racism in the air around the prevention of mixing and the preservation of “natural kinds” in activists’ rhetoric about transgenic organisms. Shiva, a woman of color from the Global South, disagreed and argued that denying subjecthood to other species, which for her inevitably happens when these boundary crossings occur, is “to converge with the approach of capitalist patriarchy” (2016: 73). While Shiva’s rhetoric can be criticized for its over-reliance on moral and political binaries, I agree that it is dangerous to equate resistance to transgenic organisms with racism among humans (see also Curry 2021; Hartigan 2017; Subramaniam 2014 for further discussions of this issue). As Shiva’s work shows, resistance to genetic engineering is not only White middle-class pearl-clutching, so Haraway might instead have foregrounded the multiple environmental injustices suffered by people of color, low-income, and Indigenous people across the world, who have also been at the forefront of resistance to the violent philosophies and practices of industrialized agriculture (see Hoover 2017; Whyte 2016).

While the binary figures of the commercial seed and the saved seed are vital to activist rhetoric, such stark contrasts belie the malleability of the boundaries between culture and nature, purity and diversity, reliability and unreliability that characterize seed-saving practice. In fact, this malleability is one of the key characteristics that seed savers value. Over generations, as their environments change, whether because they have moved, or have stayed in place but had different conditions in the climate, soil fertility, or abundance of pollinators, or because of the quality of care they have been given by their human cultivators, they incorporate their situated environments. If controlling and prioritizing seeds simply for their genetic profile denies their capacity to bring with them a “world” beyond the laboratory environment, then the very fact that seeds are malleable is erased from the identity and value of commercially bred seeds. Commercially bred seeds, then, are not only removed from their usual biosocial relations or multispecies kinships; stripping away their malleability and capacity for variation changes their very nature. It is this that seed savers are concerned about: the denaturing of seeds’ embodied relationships with their environments and, with that, their inherent malleability and inherently malleable inheritance.

## Conclusion

As Banu Subramaniam’s (2014) work shows, contesting the pure line ontology and the binaries it reinforces is complicated and onerous because it has grown deep roots. Yet, blurring the pure line, as in situ seed savers do, has radical potential for anti-capitalist and even decolonial relationships between people and plants (Graddy 2013); Subramaniam models this kind of iconoclastic mixing through her weaving together of the worlds of biology, feminist theory, and fictional and scholarly genres.

Writing about the waxing and waning of dietary advice in the United States, E. Melanie DuPuis (2015: 13) points out that, “the search for freedom as purity is a search for a world that does not exist, because we are not fixed boundaries but embedded in a world of difference that can’t help but become a part of us.” DuPuis puts forward a compelling argument for a rejection of the binaries of purity and romance that she diagnoses in Americans’ ambivalent relationship to food and the boundaries of the body. Instead, she argues for a “fermentive politics,” which is based in multispecies openness, collaboration, and trust. In this politics, which is also driven by social justice and environmental concerns, the world is let in rather than kept out and, in this way, it resonates strongly with the stance of London seed savers. Fermentation is risky, as DuPuis acknowledges and, for some, it is just too funky. But fermentation is also increasingly thought of as something that can be good for all species. Politically and ethically, it has the potential to reinscribe reciprocal relationships across species. It also holds the promise of shifting perceptions about how individuals are connected with their environments, revealing that they are only isolable with intense controls, which may, in fact, have deleterious consequences for all. This could perhaps also be described as a form of multispecies kinship: Not only does fermentation build connections of mutuality between individuals of all kinds of sizes and species, but it is based in shared substance and situs.

The political-ethical claims that seed savers make about the dangers of overreliance on an ever-decreasing range of commercially bred monocrops are highly compelling in themselves, especially in a time of environmental crisis and mass extinction. Here, I have theorized what these claims articulate about how seed savers conceptualize the nature of seeds. Seed savers value in situ conservation, openpollination, and the sharing and swapping of seeds in non-monetary transactions not only because of their political and ethical values, as important as they may be. They are also concerned that when human intervention in plant reproduction takes the form of removing seeds from their worlds, they are denatured. Seed savers are concerned about losing biodiversity through the loss of absolute numbers of plant varieties, certainly, but also through plants losing their capacity to adapt and remake themselves across generations through their complex, ongoing relationships with their environments.

But, perhaps after all, nature can bite back. The fact that F1 hybrid seeds do not reproduce true-to-type is a grave limitation if one’s income depends on producing a stable, uniform crop each year, with potentially dire consequences for people’s livelihoods and well-being. Yet another way of looking at this inability to reproduce true-to-type is to think of their inherent malleability returning with a vengeance in the second generation.
